# 382. A Hot Topic: the Relationship Between Climate Variability and Vector Specific Trends in Tick-Borne Diseases in New Jersey

**DOI:** 10.1093/ofid/ofae631.117

**Published:** 2025-01-29

**Authors:** Evelyn Wu, Bobby Brooke Herrera, Prince Baffour Tonto, Navaneeth Narayanan, Moulika Baireddy, Mathieu Gerbush, David A Robinson, Thomas Kirn, Ahmed Abdul Azim, John P Mills, Keith S Kaye

**Affiliations:** Stanford Health Care (current), Rutgers Robert Wood Johnson Medical School (past), Los Altos, California; Rutgers Global Health Institute; Rutgers Robert Wood Johnson Medical School, New Brunswick, New Jersey; Rutgers Global Health Institute; Rutgers Robert Wood Johnson Medical School, New Brunswick, New Jersey; Rutgers University Ernest Mario School of Pharmacy & Robert Wood Johnson University Hospital, New Brunswick, NJ; TIGMER/Laredo Medical Center, Laredo, Texas; Office of the NJ State Climatologist, Rutgers University, New Brunswick, New Jersey; Office of the NJ State Climatologist, Rutgers University, New Brunswick, New Jersey; Rutgers Robert Wood Johnson Medical School, New Brunswick, New Jersey; Rutgers Robert Wood Johnson Medical School, New Brunswick, New Jersey; Rutgers Robert Wood Johnson Medical School, New Brunswick, New Jersey; Rutgers Robert Wood Johnson Medical School, New Brunswick, New Jersey

## Abstract

**Background:**

The distribution and prevalence of tick-borne diseases (TBDs) are affected by climate factors, particularly temperature and humidity, which impact tick survival and activity. Understanding how climate factors affect TBDs is crucial for public health preparedness. We investigated trends in TBDs in New Jersey (NJ) over the past two decades.

**Figure 1:**
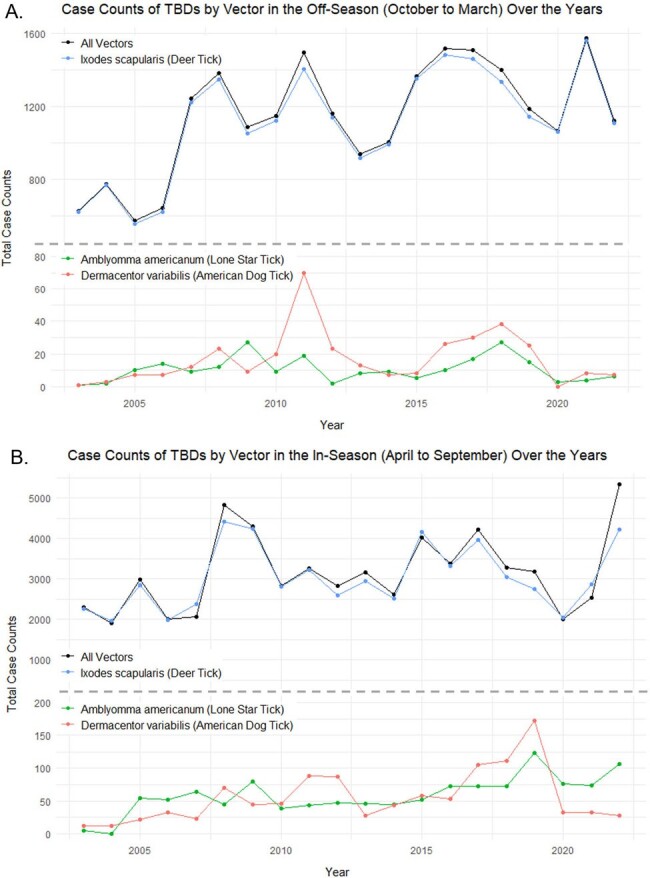
Case counts of TBDs by vector in the (A) off-season and (B) in-season months over the years (A) Case counts of reportable TBDs in aggregate and diseases transmitted by I. scapularis significantly increased in the off-season. Diseases transmitted by A. americanum and D. variabilis did not significantly increase in the off-season. (B) Case counts of all reportable TBDs, and diseases transmitted by I. scapularis and D. variabilis did not significantly increase in the in-season. In contrast, diseases transmitted by A. americanum significantly increased in the in-season.

**Methods:**

We obtained case counts of reportable TBDs from 2003 to 2022 from the NJ Department of Health (N = 85,905), and climate data from the publicly available nClimDiv dataset. We conducted linear regression analysis to assess temporal trends in total TBD cases over the 20-year period. We also used Poisson regression analysis to examine the association between TBD incidence and climate factors, specifically average temperature and total precipitation.

**Table 1:**
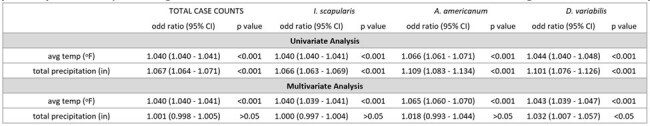
Case count correlation with climate parameters (average temperature and total precipitation) using univariate and multivariate Poisson regression analysis

We performed a Poisson regression analysis using case count as the dependent variable, and average temperature and total precipitation as the independent variables. For the multivariate model, we utilized average temperature and total precipitation as covariates. Total case counts of reportable tick-borne diseases, and case counts of diseases transmitted by I. scapularis (Lyme disease, Babesiosis, Anaplasmosis), A. americanum (Ehrlichiosis), and D. variabilis (Spotted Fever Group Rickettsiosis) are significantly positively correlated with average temperature and total precipitation using univariate analysis. Only diseases transmitted by the American Dog Tick are positively correlated with both average temperature and total precipitation using multivariate analysis. Diseases transmitted by I. scapularis and the lone star tick are only positively correlated with average temperature using multivariate analysis.

**Results:**

Total TBDs have significantly increased over the study period, particularly during the off-season (October to March; p < 0.01), while there was no significant increase during the in-season period (April to September) (Fig. 1, p > 0.05). Sub-analysis by vector revealed significant increases in diseases transmitted by *Ixodes scapularis* (Deer Tick; Babesiosis, Lyme disease, Anaplasmosis), particularly in the off-season (Fig. 1A; p < 0.01). In contrast, diseases transmitted by *Amblyomma americanum* (Lone Star Tick; Ehrlichiosis) significantly increased during the in-season period (Fig. 1B; p < 0.001). There was no significant increase in diseases transmitted by *Dermacentor variabilis* (American Dog Tick; Spotted Fever Group Rickettsiosis) (Fig. 1; p > 0.05). Univariate Poisson regression analysis showed a positive correlation between all TBDs and average temperature and total precipitation (Table 1).

**Conclusion:**

Our findings demonstrate a rising trend in TBDs in NJ, correlated with average temperature and precipitation. Sub-analysis highlights vector-specific patterns, with *I. scapularis-*transmitted diseases increasing predominantly in the off-season and *A. americanum-*transmitted diseases during the in-season period. These findings emphasize the importance of maintaining vigilance for TBDs during traditionally low-risk months and the need for proactive strategies to mitigate the impact of climate factors on TBD transmission.

**Disclosures:**

**Bobby Brooke Herrera, PhD**, Mir Biosciences, Inc.: co-founder **Navaneeth Narayanan, PharmD, MPH, BCIDP**, Astellas: Honoraria|Beckman Coulter: Honoraria|Merck: Grant/Research Support|Shionogi: Grant/Research Support **Thomas Kirn, MD PhD**, Selux Diagnostics: Advisor/Consultant|Selux Diagnostics: Honoraria **Keith S. Kaye, MD, MPH**, Allecra: Advisor/Consultant|CARB-X: Advisor/Consultant|GSK: Advisor/Consultant|Merck: Advisor/Consultant|Shionogi: Advisor/Consultant|Spero: Advisor/Consultant

